# Fabrication and assessment of performance of clay based ceramic membranes impregnated with CNTs in dye removal

**DOI:** 10.1038/s41598-024-77015-3

**Published:** 2024-11-05

**Authors:** Kareem H. Hamad, Heba Abdallah, Sohair T. Aly, R. Abobeah, Sh. K. Amin

**Affiliations:** 1 Egyptian Academy for Engineering and Advanced Technology (EA&EAT) Affiliated to Ministry of Military Production, Cairo, Egypt; 2 Chemical Engineering and Pilot Plant Department, Engineering and Renewable Energy Research Institute, National Research Centre (NRC), Dokki, Giza, Egypt; 3 Department of Chemical Engineering, Faculty of Engineering, El-Minia University, El-Minia, Egypt

**Keywords:** Wastewater treatment, Ceramic membranes, Surface modification, Carbon nanotubes, Environmental sciences, Engineering

## Abstract

In this research, flat disk clay-based ceramic membranes were fabricated and optimized for use in the treatment of wastewater contaminated with dye. The properties of the fabricated membranes were assessed to optimize the fabrication conditions, namely, the firing temperature (1150 °C, 1200 °C, and 1250 °C), soaking time (30 min and 60 min) and zeolite percentage (0%, 10%, and 20%). On the other hand, the rejection of methylene blue dye (MB) and acid fuchsin dye (AF) was studied. The surface of the optimal membrane support was modified using functionalized COOH-carbon nanotubes to increase the dye removal percentage. The fabricated membranes were characterized using FTIR, XRD, and XRF. The optimum membrane support was fabricated at 1150 °C, after 30 min of soaking and with 0% zeolite. The most suitable membrane support was found to be AF, as its rejection percentages reached 42% and 95% without and after surface modification, respectively. The surface of the membrane was examined via SEM, which revealed normally distributed pores. The average pore size of the final membrane was found to be 0.076 micrometers using a mercury porosimeter; thus, the produced membranes can be used in ultrafiltration applications. Finally, the fouling properties showed that the total fouling reached 72.8%, of which only 2.1% was irreversible.

## Introduction

Water, food, and energy are the main pillars to achieve sustainability, the water crisis is one of the most important topics. According to the World Health Organization, more than two billion people live in water-stressed regions^1^. Therefore, water treatment techniques have gained importance and have attracted researchers to lower the cost of such techniques in addition to increasing their efficiency and maximizing their range of applicability. Industrial wastewater is discharged in large amounts and has a negative impact on the environment and on elements of life (humans, plants, and animals). Industrial wastewater contains different types of pollutants; depending on the source and industrial activity, wastewater may contain different organic and inorganic pollutants^2,3^. One of the most hazardous wastewater types is contaminated with dye due to its hazardous and carcinogenic properties even at low concentrations^4,5^. In 2019, dye and pigment consumption reached approximately 7.5 million tons, with an estimated two hundred and eighty thousand tons of dye discharged each year in industrial wastewater^6,7^.

Dyes are considered one of the major pollutants to the water environmental ecosystem and to human health; they have diverse negative effects on many human organs, including the skin, kidney, and liver. On the other hand, when dyes are disposed, they prevent light from penetrating, and as a result, photosynthesis is affected. Not only that, but also when degradation of dyes takes place, it consumes dissolved oxygen in the water, thus affecting the aquatic system^8^.

The global energy consumption is increasing rapidly, the International Energy Agency (IEA) predict that by 2050 the energy consumption will increase by 27%. The increase in energy consumption will result a huge stress on the energy resources, thus new technologies are emerging to offer a highly efficient yet low energy consumption solutions^9^.

Different treatment techniques, such as distillation, crystallization, ion exchange, adsorption, and membrane filtration, have been used for dye removal from wastewater^10^; however, one of the promising techniques is membrane filtration due to its advantages, which surpasses its disadvantages^11,12^, membrane technologies offer a low energy demand technology that could achieve high separation efficiency with minimal environmental impact^9^. One of the most common types of membranes is ceramic membranes, which are more stable than polymeric membranes due to their mechanical strength, chemical stability, wide operating conditions, and longer life expectancy^13,14^, on the other hand, it has disadvantages of their low flexibility, high cost, and limited pore sizes^15^. Different materials can be used in the preparation of ceramic membranes, one of the most commonly used materials is clay, as it has a low cost and unique physicochemical properties. It was found that clay minerals contribute to the formation of narrow pores, thus increasing the separation of the membrane^16–19^. On the contrary, the main disadvantage of clay-based membranes is their low mechanical strength^20^, that is why many research are conducted to find the optimum fabrication conditions and blends to achieve both acceptable physical properties and highest performance.

Different materials were used in the fabrication of ceramic membranes, the most dominant low-cost materials are clays, and kaolin followed by fly ash then other materials such as natural zeolites, and geopolymers, etc., these materials offer a cheap membrane that has high efficiency, as a result ceramic membranes could be produced for large scale applications. Several research studies reported the efficient use of low-cost ceramic membranes in various applications such as removal of suspended solids, oil droplets, dyes, bacteria, and heavy metals^21^.

Zeolites are crystalline materials that can be synthesized or obtained from nature. Zeolites are composed mainly of silicon, aluminum and oxygen, it has been used widely in many research as its usage in membranes allows only lower size particles to pass^22^, in addition to the enhancement of chemical and thermal stabilities of membranes^23,24^. Blends of zeolites and clay show better mechanical strengths at lower sintering temperatures, besides lowering the shrinkage of clay-based membranes^25^.

Modification of clay-based ceramic membranes with nanomaterials enhances contaminant removal ability, and several studies have used different nanomaterials, such as titanium dioxide^26–28^, silver^29^ and carbon nanotubes^30,31^. Carbon nanotubes (CNTs) have attracted researchers due to their promising performance and properties. CNTs can be directly incorporated by mixing them with different materials during the fabrication of ceramic membranes, or they can be deposited on the surface of ceramic membranes^32,33^. Functionalized CNTs have distinguished properties, such as high mechanical strength and large surface area, and are used to produce membranes with higher selectivity and better fouling resistance than other materials^34^.

In this study, a clay based ceramic membrane is fabricated. Then, an assessment of zeolite addition was carried taking into consideration production variables including sintering temperature, and soaking time. The optimum support was selected based on its physical properties, performance, and economical aspects, taking into consideration that three membranes were used to get the average result of each property. Finally, The optimum membrane was impregnated by CNTs to increase its performance and enhance its antifouling characteristics.

## Experimental

### Materials

Ball clay was used as a source of silica and alumina, and starch was used as a pore-forming agent; both of these materials were purchased from local markets. Polyvinyl alcohol was used as a binder purchased from Alpha Chemicals, as was zeolite, methylene blue, and acid fuchsin dye. Functionalized COOH-Carbon Nanotubes (COOH-CNTs) were used for surface modification. These materials were purchased from the Grapen Turkey supplier and had a purity of 93.8%, a specific surface area of 25 m^2^/g and a density of 215 kg/m^3^.

### Characterization of raw materials

Physical analysis of the raw materials was performed to determine the particle size distribution and particle density.

The screen analysis was performed according to ASTM E11-2022^35^, and the particle density was determined using a pycnometer. The equation used is shown below, where m_o_ is the mass of the empty pycnometer (g); m_s_ is the total mass of the pycnometer filled with solid powder only (g); m_l_ is the mass of the pycnometer filled with distillate water only (g); m_sl_ is the total mass of the pycnometer filled with solid powder and distillate water (g); ρ_s_ is the density of solid particles (g/cm^3^); and ρ_w_ is the density of water (g/cm^3^).

1 $${\mathrm\rho}_{\mathrm s}=\frac{\left({\mathrm m}_{\mathrm s}-{\mathrm m}_{\mathrm o}\right)\ast{\mathrm\rho}_{\mathrm w}}{\left({\mathrm m}_{\mathrm l}-{\mathrm m}_{\mathrm o}\right)-\left({\mathrm m}_{\mathrm{sl}}-{\mathrm m}_{\mathrm s}\right)}$$

#### X-ray diffraction (XRD)

The study was performed at the Housing and Building National Research Center (HBRC) using an X’pert Pro PANalytical instrument manufactured by Panalytical B. V Co.,

#### XRF

The XRF test was performed at the Housing and Building National Research Center (HBRC). The XRF instrument used was an Axio sequential spectrometer manufactured by PANalytical, Netherlands.

#### Thermal gravimetric analysis

This analysis was performed using thermogravimetric analysis (TGA, Shimadzu DTG-60, Japan) with an STA 6000 Perkin Elmer Analyzer from 25 °C to 900 °C at a heating rate of 10 °C/min under argon.

#### Attenuated total reflection–FTIR (ATR-IR)

ATR-IR was performed with a range of 400–4000 cm^−1^ by an ALPHA II BRUKER, USA.

### Preparation of the ceramic membrane

The pressing technique was used for the production of disk membranes. First, a PVA solution was prepared by adding 3 wt% PVA slowly to water at a temperature of 88–90 ^o^C, and mechanical mixing was performed for around 20 min on a hot plate until complete dissolution was achieved. Afterwards, the solution was allowed to cool. Clay was ground, and 5 wt% starch was added as a pore-forming agent along with 15 wt% polyvinyl alcohol solution to bind the mixture.

**Figure 1:**
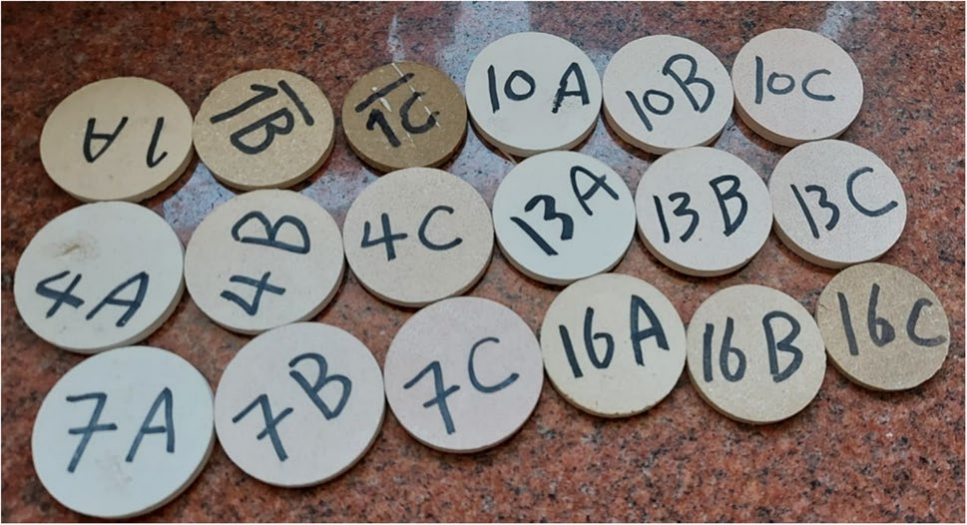
Fabricated membranes after coding.

The mixture was compressed at 2 tons using a hydraulic pressing machine, after which the membrane was left in an oven at three different temperatures (1150 °C, 1200 °C, and 1250 °C) for different soaking times (30–60 min) to obtain disks 5 cm in diameter and 0.5 cm in thickness, these ranges were chosen according to literature as they have direct effect on different physical specifications such as compressive strength and density^15,36,37^. The membranes were dried at 60 °C for 1 h and then at 100 °C for another hour, after which the membranes were left at ambient temperature overnight to ensure complete dryness of the membrane. The sintering was carried out in a furnace as the cycle started at room temperature and continued at 500 °C, after which the samples were incubated at this temperature for an hour to ensure the removal of volatile matter (ex. organics), then held at 500 °C to 950 °C, held for an hour to enable decomposition of carbonates and dihydroxylation of clay minerals, held at 950 °C to the final temperature and held at that temperature for a specific soaking time to enable densification of membranes^18^. The rate of heating throughout the cycle was 5 °C/min to minimize thermal stresses^13^. After the membranes are produced, the membranes are labeled as shown in Fig. 1. The raw materials undergo different physical, chemical, and thermal analyses; on the other hand, the produced membranes undergo different tests in addition to an assessment of their performance. According to the results, the optimum membrane was identified and impregnated with CNTs to increase its performance.

### Characterization of the membranes

The physical properties of the produced membranes were calculated, such as the bulk density, which was calculated based on the ASTM C373-18 (2023) standard method^38^; cold water absorption, which was determined based on the ASTM C67-21^39^ standard method; apparent porosity, which was calculated according to ASTM C373-14^40^; and compressive strength, which was determined on cubes 5 × 5 × 5 cm that match the conditions of fabrication of each membrane according to ASTM C67-21^39^.

### Membrane properties and application

The performance of the membrane was assessed by dynamic filtration, after which the optimum membrane properties were assessed, as discussed below.

#### Dynamic filtration

**Figure 2:**
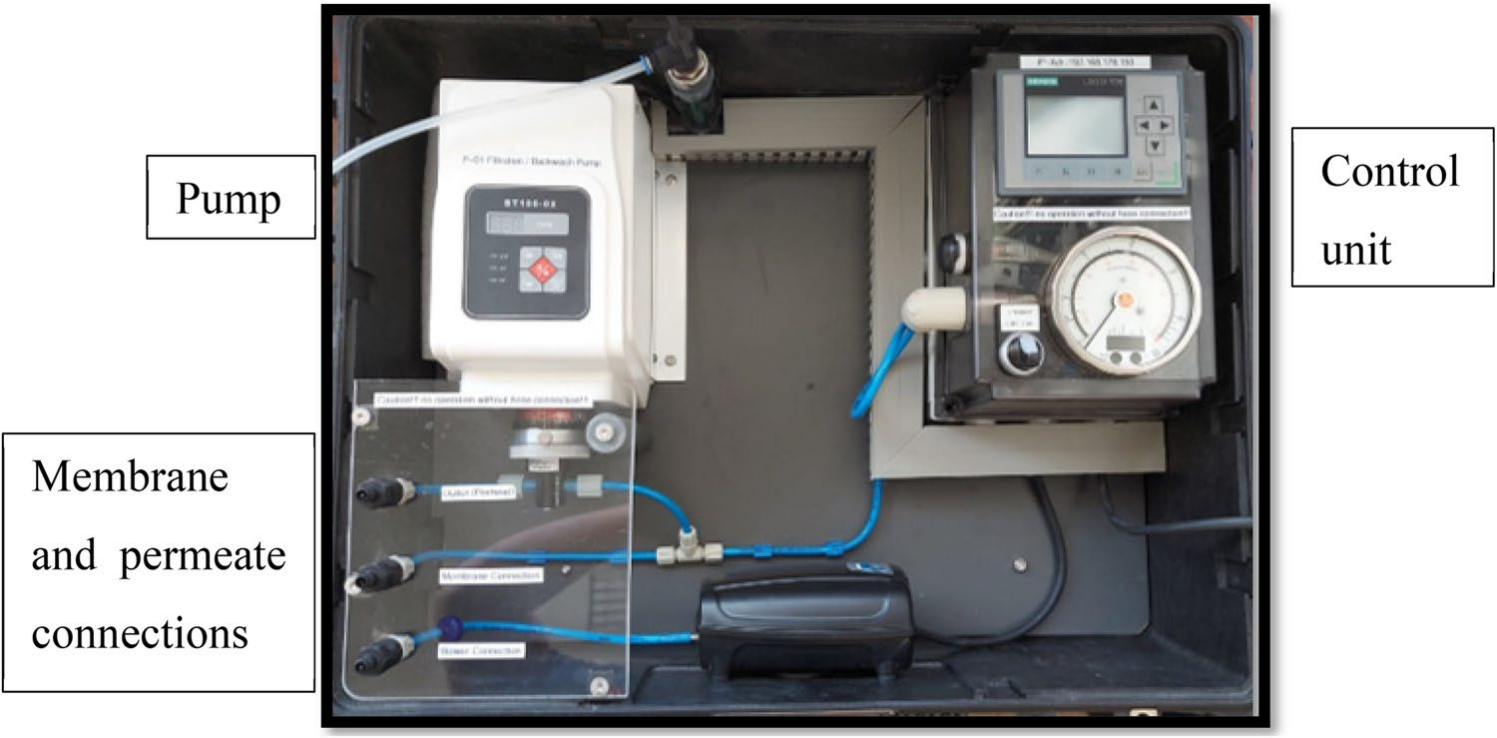
Siemens cerafiltec device.

A Siemens cerafiltec device was used to perform the tests on the membranes. The device shown in Fig. 2 contains a pump that forces water to pass through the membrane placed inside the compartment; thus, filtration was achieved, and different tests could be performed. The water was pumped at a constant flux of 150 L/m^2^h, and a sample was taken after 30 min for analysis via UV spectroscopy.

#### Flux

The water flux (L/m^2^.h) was calculated by dividing the amount of water that passed through the membrane at a certain time by the area of the membrane.

2 $$\mathrm{Flux}=\frac{\mathrm{water}\;\mathrm{passed}}{\mathrm{Area}}$$

#### Dye removal

To determine the percentage of dye removal, ultraviolet spectroscopy was used, a calibration curve was calibrated for each dye, 100 PPM synthetic dye wastewater was prepared, the concentration of dye in each water sample was assessed before and after passing through the membrane, and the removal percentage was calculated by

3 $$\mathrm R\%=\frac{\mathrm{Initial}\;\mathrm{concentration}-\mathrm{Final}\;\mathrm{concentration}}{\mathrm{Initial}\;\mathrm{concentration}}$$

#### Fouling

The resulting membrane antifouling properties were studied by calculating the reversible and irreversible fouling. First, the membrane was subjected to pure water for 1 h, sample collection was performed at 15-minute intervals, and the final pure water flux (Jw,1) (L/m^2^.h) was calculated using the following equation:

4 $$\mathrm{Jw},1=\frac{\mathrm{volume}\;\mathrm{of}\;\mathrm{water}\;\mathrm{passed}}{\mathrm{Area}\ast\mathrm{Time}}$$

Then, the membrane was subjected to a solution of 100PPM dye and 100PPM humic acid for an hour, the total flux was calculated (Jp) in (L/m^2^.h), and the flux at 15-minute intervals was calculated. Finally, the membrane was backwashed with pure water for 30 min, after which the pure water flux was calculated for another hour at 15-minute intervals (Jw,2) (L/m^2^.h). The flux recovery ratio (FR) was calculated as follows:

5 $$\mathrm{FR}\%=\frac{\mathrm{Jw},2}{\mathrm{Jw},1}\ast100$$

Reversible fouling represents the percentage of fouling that could be removed by hydraulic backwash; irreversible fouling is the percentage of fouling that could not be removed and can be calculated using following equations:

6 $$\mathrm{Rr}\%=\frac{\mathrm{Jw},2-\mathrm{Jp}}{\mathrm{Jw},1}\ast100$$

7 $$\mathrm{Rir}\%=\frac{\mathrm{Jw},1-\mathrm{Jw},2}{\mathrm{Jw},1}\ast100$$

The total loss of permeation due to both reversible and irreversible fouling can be calculated using the following equation:

8 $$\mathrm{Rt}\%=\frac{\mathrm{Jw},1-\mathrm{Jp}}{\mathrm{Jw},1}\ast100$$

#### Field emission scanning electron microscopy (FESEM)

The surface of the membrane was investigated using an FESEM QUANTA 250 (Japan). A magnification of 3kx was used at a potential difference of 10 keV to examine the surface of the optimum ceramic membrane before and after surface modification.

#### Pore size distribution

The pore size distribution, total porosity, and average pore diameter were examined according to ISO 15901-1:2016^41^ using the mercury porosimeter PORESIZER 9320.

## Results and discussion

### Raw material characterization

#### Screen analysis

**Figure 3:**
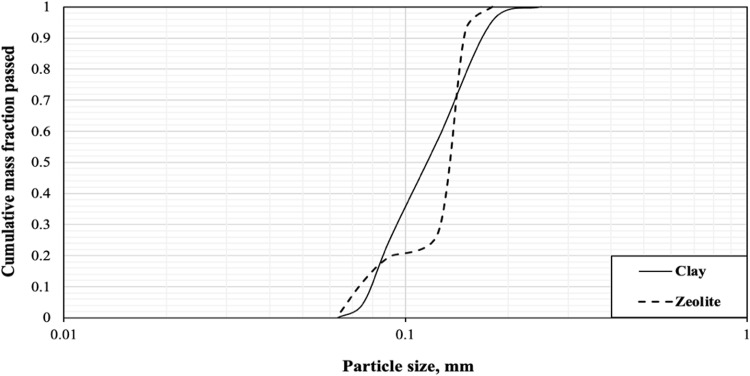
Cumulative mass fraction passed for different raw materials.

Screen analysis was performed using a set of screens with the previously mentioned opening sizes. The cumulative mass fractions are plotted against the particle diameter to obtain the median particle diameter, and the median particle sizes of the clays are 115 μm and 145 μm for zeolite as shown in Fig. 3.

#### Powder density

The densities of the clay and zeolite were obtained by a pycnometer, and the reference fluid used was distilled water. The densities are 1.625 g/cm^3^ and 1.75 g/cm^3^ for clay and zeolite, respectively.

#### XRF

As obtained from the XRF results, the clay contained mainly silica and alumina, which are considered the main compounds. Silica, alumina, iron oxide, magnesium oxide and calcium oxide represent approximately 75% of the total mass of the sample, and the percentage of volatile matter that evaporates upon ignition (L.O.I.) is 21.93%. On the other hand, zeolite mainly contains oxides of silica and alumina, accounting for approximately 79.33%. and the loss on ignition is 12.37%. The analysis of the raw materials is tabulated in Table 1.

**Table 1:** Different constituents in clay and zeolite.

Percentage (%) Constituents	Clay	Zeolite
SiO_2_	41.08	67.22
Al_2_O_3_	21.72	12.11
Fe_2_O_3_	0.68	2.42
CaO	7.51	1.56
MgO	4.73	0.48
Na_2_O	0.24	0.88
K_2_O	0.88	2.31
SO_3_	0.21	0.02
TiO_2_	0.54	0.2
P_2_O_5_	0.06	0.02
ZrO_2_	0.032	0.097
Cr_2_O_3_	0.012	---
SrO	0.018	0.029
MnO	---	0.097
BaO	0.241	0.078
ZnO	0.021	0.021
Cl^−^	0.03	0.05
Rb_2_O	0.011	0.014
Y_2_O_3_	---	0.01
Nb_2_O_5_	---	0.017
NiO	0.005	---
PbO	0.013	---
Co_3_O_4_	0.015	---
L.O.I	21.93	12.37
Total	99.978	100

#### XRD

**Figure 4:**
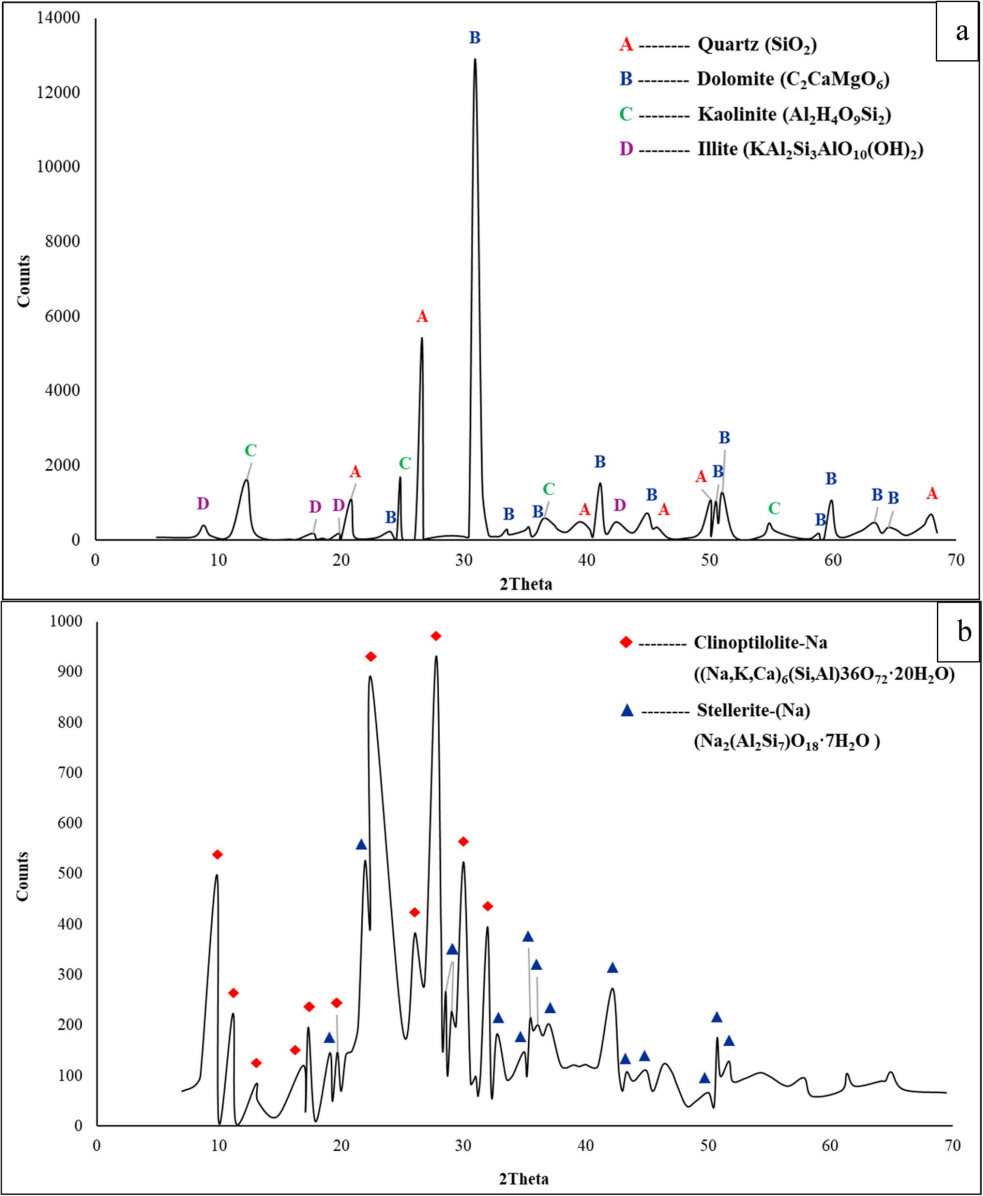
XRD patterns of (**a**) clay and (**b**) zeolite.

The results from the XRD test show that the main crystalline phases present in clay are quartz, dolomite, kaolinite and illite, while for zeolite, the main phases are clinoptilolite-Na and stellerite-(Na), as shown in Fig. 4. The resulting phases are compatible with the XRF results, as they represent different combinations of the elements present in each sample. The chemical formula of each phase is tabulated in Table 2. Like any material extracted from the Earth’s crust, clay may contain different combinations of items according to the type and origin of the rocks. Thus, quartz (SiO_2_), kaolinite (SiO_2_.Al_2_O_3_.2H_2_O), dolomite, and illite are found in clay^42^. On the other hand, zeolite consists mainly of sodium-containing phases such as clinoptilolite-Na and stellerite-(Na)^23^.

**Table 2:** Chemical formulas of the different phases in the raw materials.

Material	Phase	Chemical composition
Clay	Quartz	SiO_2_
	Dolomite	C_2_CaMgO_6_
	Kaolinite	Al_2_H_4_O_9_Si_2_
	Illite	KAl_2_Si_3_AlO_10_(OH)_2_
Zeolite	Clinoptilolite-Na	(Na, K,Ca)_6_(Si, Al)36O_72_·20H_2_O
	Stellerite-(Na)	Na_2_(Al_2_Si_7_)O_18_·7H_2_O

#### Thermal analysis

**Figure 5:**
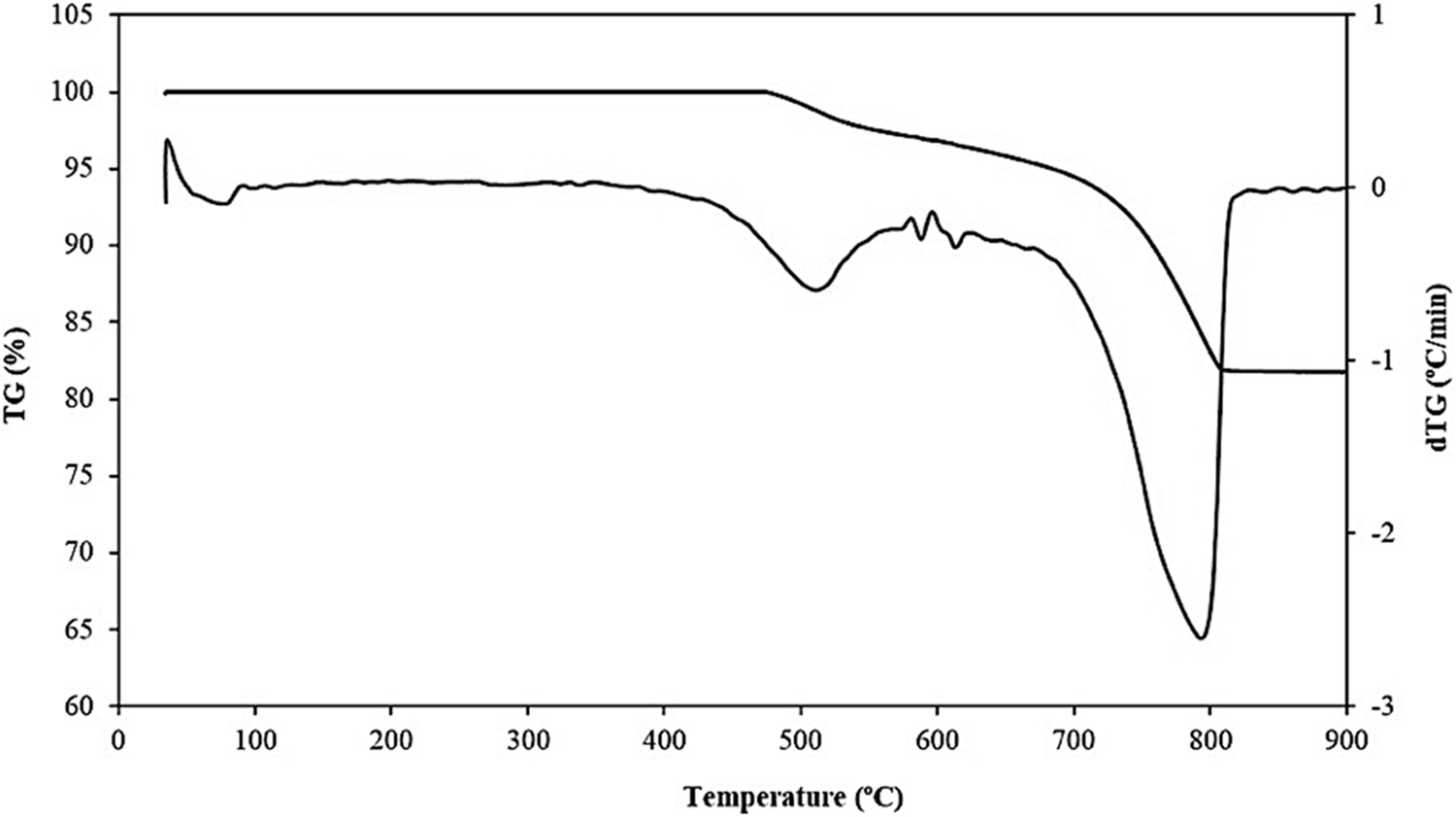
TGA of Clay.

As shown in Fig. 5, as the weight of the clay decreases, the total loss is considered to be approximately 17% of the total sample weight. This decrease occurred over two temperature ranges due to the loss of chemical water at 500 °C and the decomposition of calcium carbonate at approximately 800 °C.

**Figure 6:**
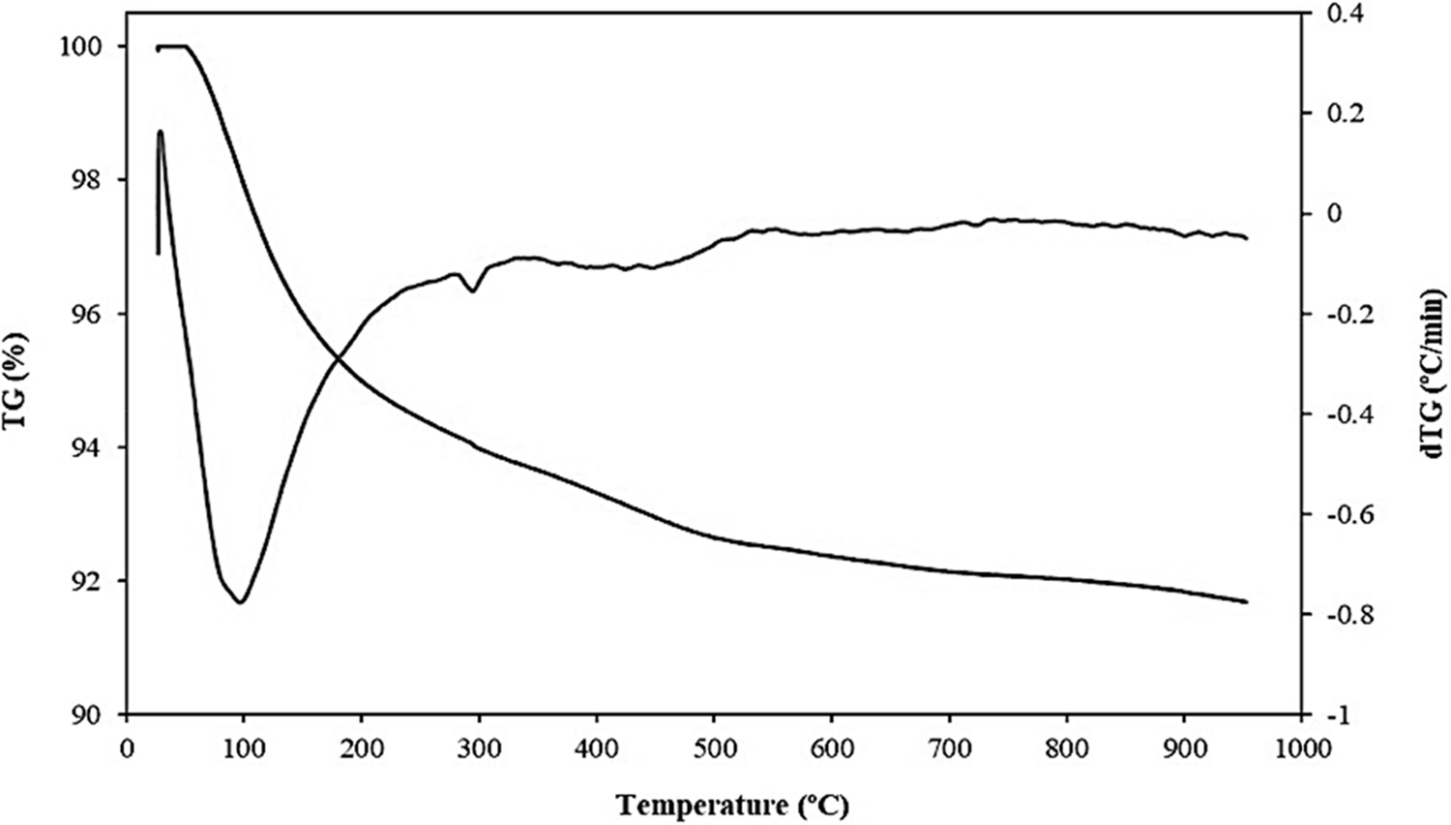
TGA of Zeolite.

The thermal decomposition of zeolite is shown in Fig. 6, which reveals that it is approximately 8% at 100 °C due to the loss of water.

**Figure 7:**
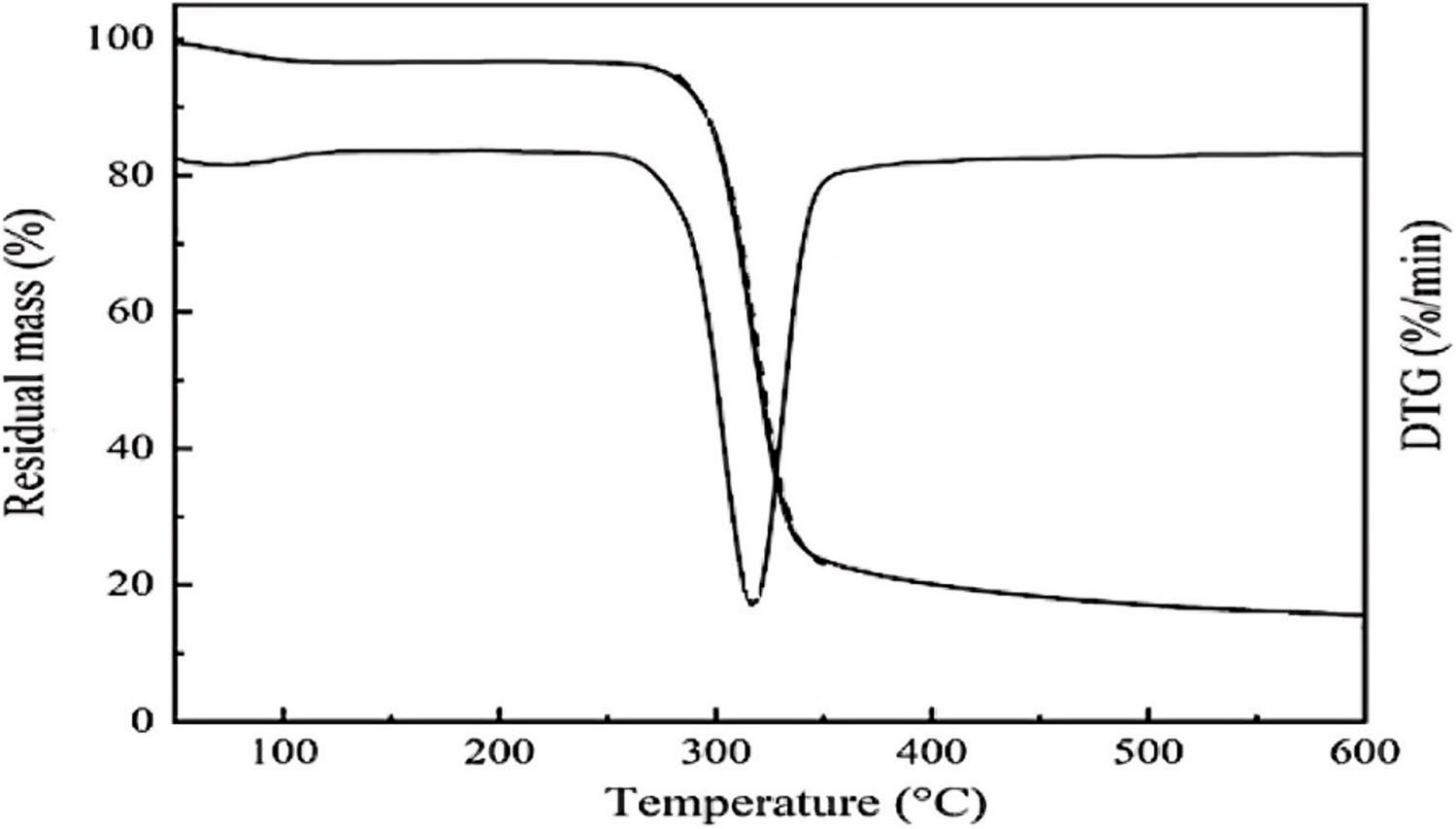
TGA of corn starch.

Figure 7 shows the TGA results for corn starch, from which it was found that the main decomposition occurred at 297 °C due to the dehydration of the alpha 1,4 glycosidic bond.

### Fourier transform infrared spectroscopy (FTIR)

**Figure 8:**
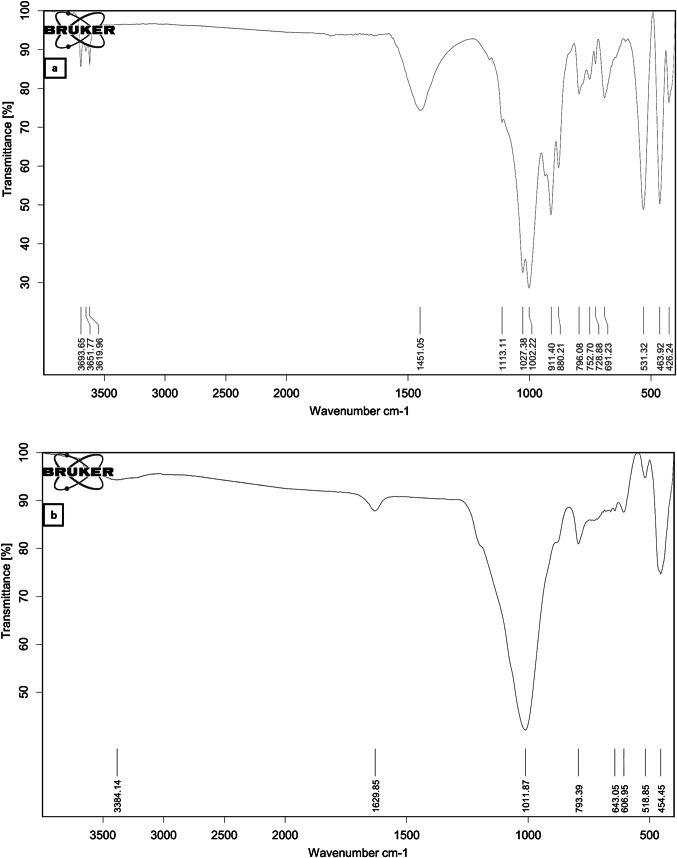
FTIR results of (**a**) clay and (**b**) zeolite.

The FTIR of both clay and zeolite are shown in Fig. 8, the FTIR of clay revealed OH stretching vibrations at wavenumber 3619.96–3694.65 cm-1, a carbonate stretching vibration at 1451.05 cm-1, Si–O stretching vibrations at 1002.22–1113.11 cm-1, a bending vibration of inner OH in the Al–OH-Al group at 911.4 cm-1, a carbonate bending vibration at 880.21 cm-1, stretching vibrations of Si-O and Si-O-Al at 796.08–691.23 cm-1, stretching vibrations of Si-O-Al at 531.32 cm-1, a bending vibration of Si-O at 463.92 cm-1, and Si-O bending vibrations at 426.24 cm-1; on the other hand, the FTIR result of zeolite showed H–O–H bending vibrations at 1629 cm-1, Si–O stretching vibrations at 1011.87 cm-1, Si-O-Al stretching vibrations at 793.39 cm-1, symmetrical stretching of the external linkage at 606.95–643.05 cm-1, Si–O–Al stretching vibrations, and Si–O–Si^43–49^.

### Ceramic membrane properties

#### Physical properties

##### Bulk density

**Figure 9:**
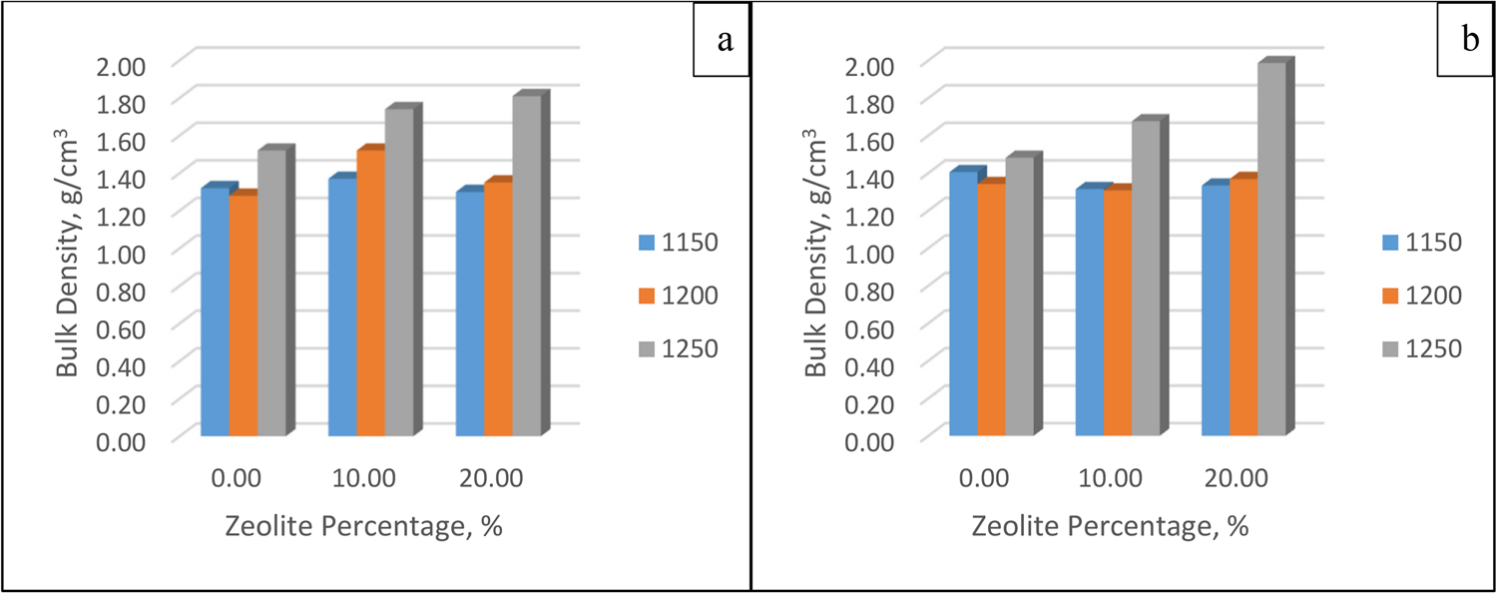
Bulk density at different zeolite percentages and temperatures after soaking for (**a**) 30 min and (**b**) 60 min.

The bulk density can be obtained for each membrane by knowing both its dry mass and its bulk volume. Figure 9 shows the relationships between the bulk density and zeolite percentage at different firing temperatures after 30 and 60 min. Clearly, as the temperature increases, the bulk density increases due to the decrease in volume. On the other hand, the zeolite percentage is directly proportional to the BD, and the bulk density increases with the zeolite percentage due to the high shrinkage of the membranes at a high zeolite percentage. On the other hand, the soaking time had a minimum effect, as indicated by the fact that the membrane nearly reached its final volume after 30 min.

##### Cold water absorption

**Figure 10:**
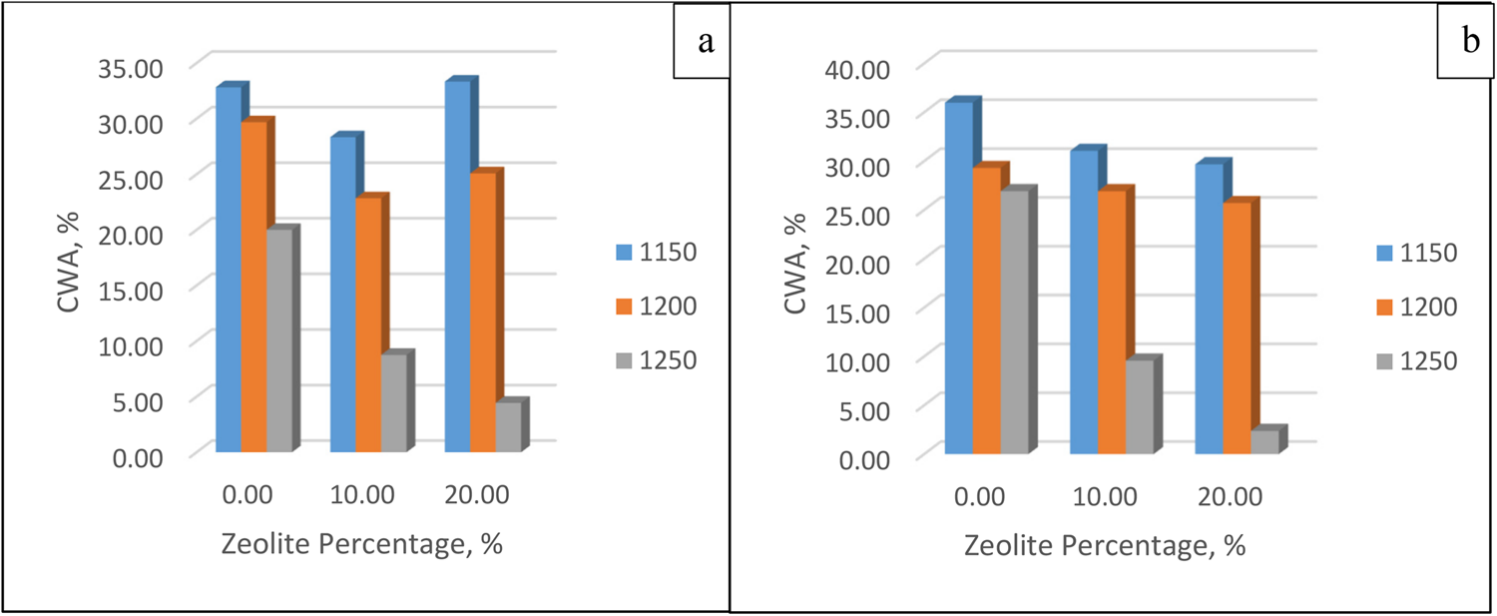
Cold water adsorption for different zeolite percentages and different temperatures at soaking times of (**a**) 30 min and (**b**) 60 min.

After the membranes were left in cold water for 24 h, the wet blocks were weighed. Then, Eq. (3.3) is applied to obtain the absorption percentage of cold water. The values and ranges of absorption percentages are shown clearly in Fig. 10 for 60 min and 30 min. It is concluded that when the temperature increases, the percentage of water absorbed decreases as the natural drying efficiency increases, leaving more pores. An increase in the zeolite percentage decreases the CWA as more clay is present in the membrane, increasing adsorption of water occurs, and soaking time does not have a significant effect on the CWA.

##### Boiling water absorption

**Figure 11:**
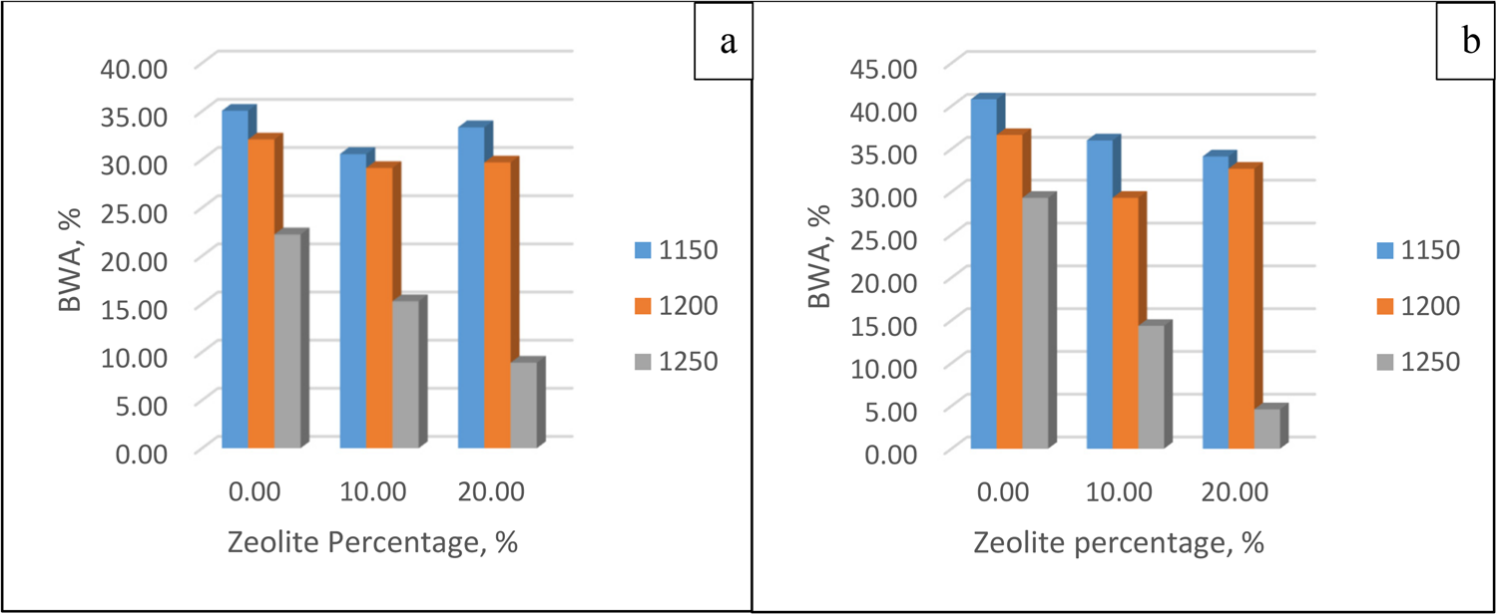
Boiling water adsorption for different zeolite percentages and different temperatures after soaking for (**a**) 30 min and (**b**) 60 min.

The membranes left in cold water for 24 h were put into boiling water for 5 h and then left at ambient temperature to heat. After being weighted, Eq. (3.4) was used to determine the absorption percentage of the boiling water. The results from this equation are plotted against zeolite percentage for different temperatures at soaking times of 30 min and 60 min, as shown in Fig. 11. It is clear that the BWA absorption decreases with increasing temperature due to the glass effect, which decreases the number of pores. These results validate the proportionality between both bulk density and water absorption, as when the bulk density increases, the specimens exhibit minimum voids, which leads to a decrease in water absorption and vice versa.

##### Apparent porosity

**Figure 12:**
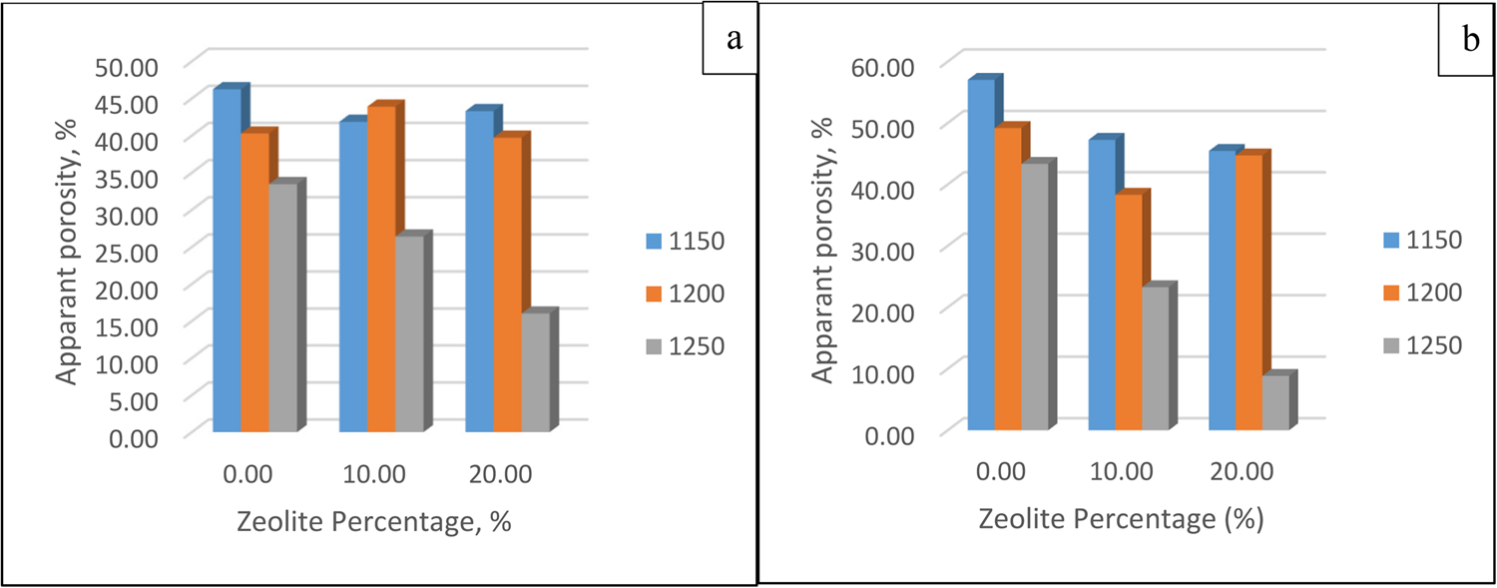
Apparent porosities for different zeolite percentages and different temperatures after soaking for (**a**) 30 min and (**b**) 60 min.

The obtained values are shown in Fig. 12. The sintering temperature significantly affects the apparent porosity, which is inversely proportional to the sintering temperature. As the sintering temperature increases, a denser structure is obtained, and a glassy phase is present. The viscous glass phase enters the pores and reduces the porosity.

##### Saturation coefficient

**Figure 13:**
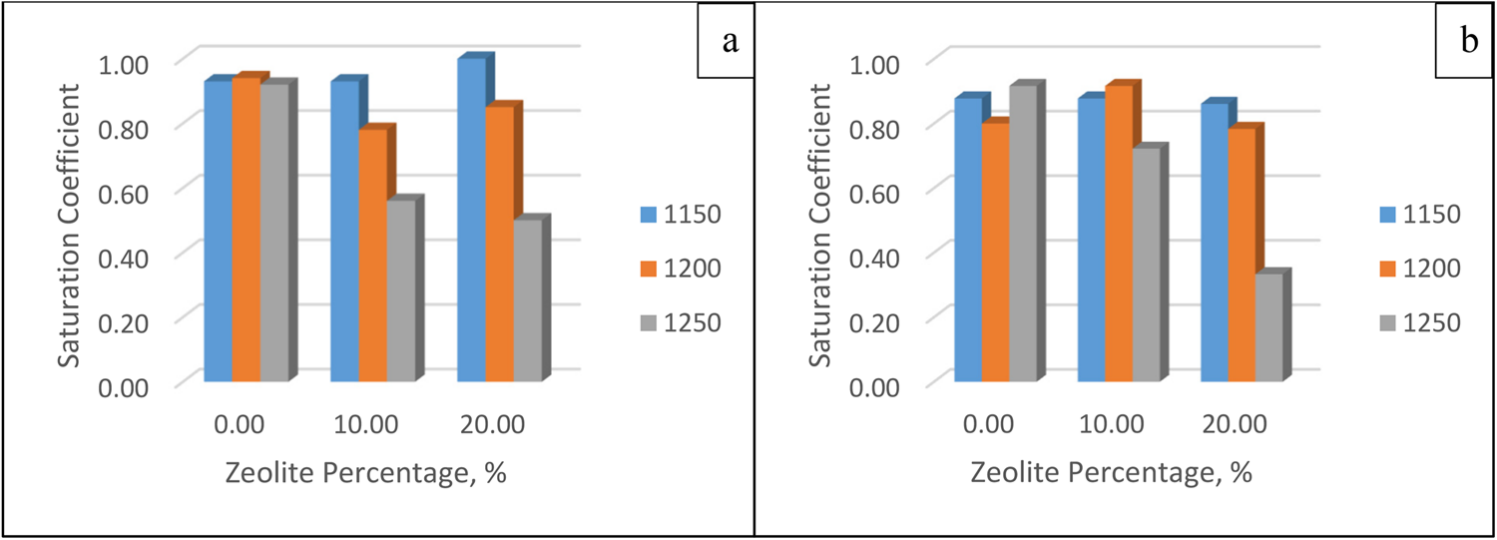
Saturation coefficient for different zeolite percentages and different temperatures after soaking for (**a**) 30 min and (**b**) 60 min.

The saturation coefficient was calculated using Eq. (3.6): The results are shown in Fig. 13. As long as the temperature increases, the saturation coefficient decreases; as the zeolite percentage increases, the saturation coefficient decreases, which is the same effect as soaking time.

##### Compressive strength

**Figure 14:**
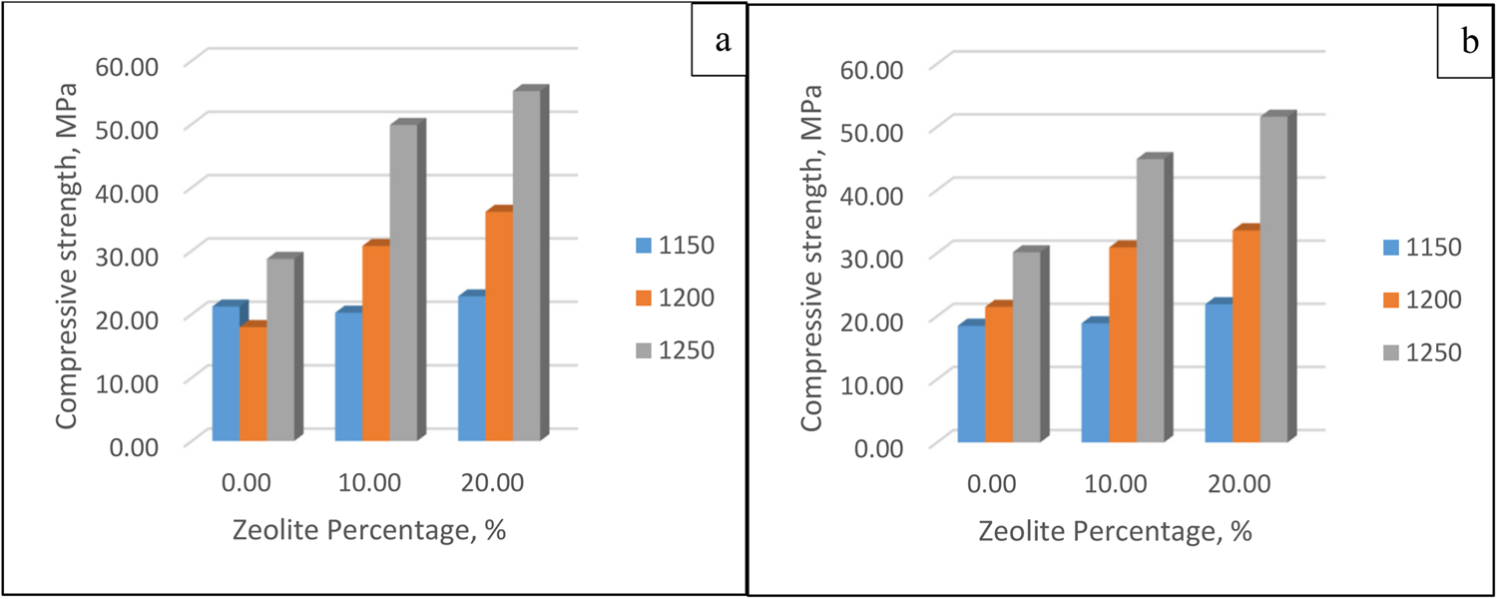
Strength for different zeolite percentages and different temperatures at soaking times of (**a**) 30 min and (**b**) 60 min.

This figure shows how the membrane could withstand the applied pressure. The strength at different temperatures, zeolite percentages, and soaking times are shown in Fig. 14. Increasing the percentage of zeolite increases the strength as the zeolite contains reactive silica and alumina in high proportions, which react with calcium hydroxide to form calcium silicate hydrate and calcium aluminate hydrate gels. On the other hand, time has a minimal effect, which means that the membrane reaches its final strength and that excess soaking time is not needed.

#### Membrane performance

##### Dye removal percent

**Figure 15:**
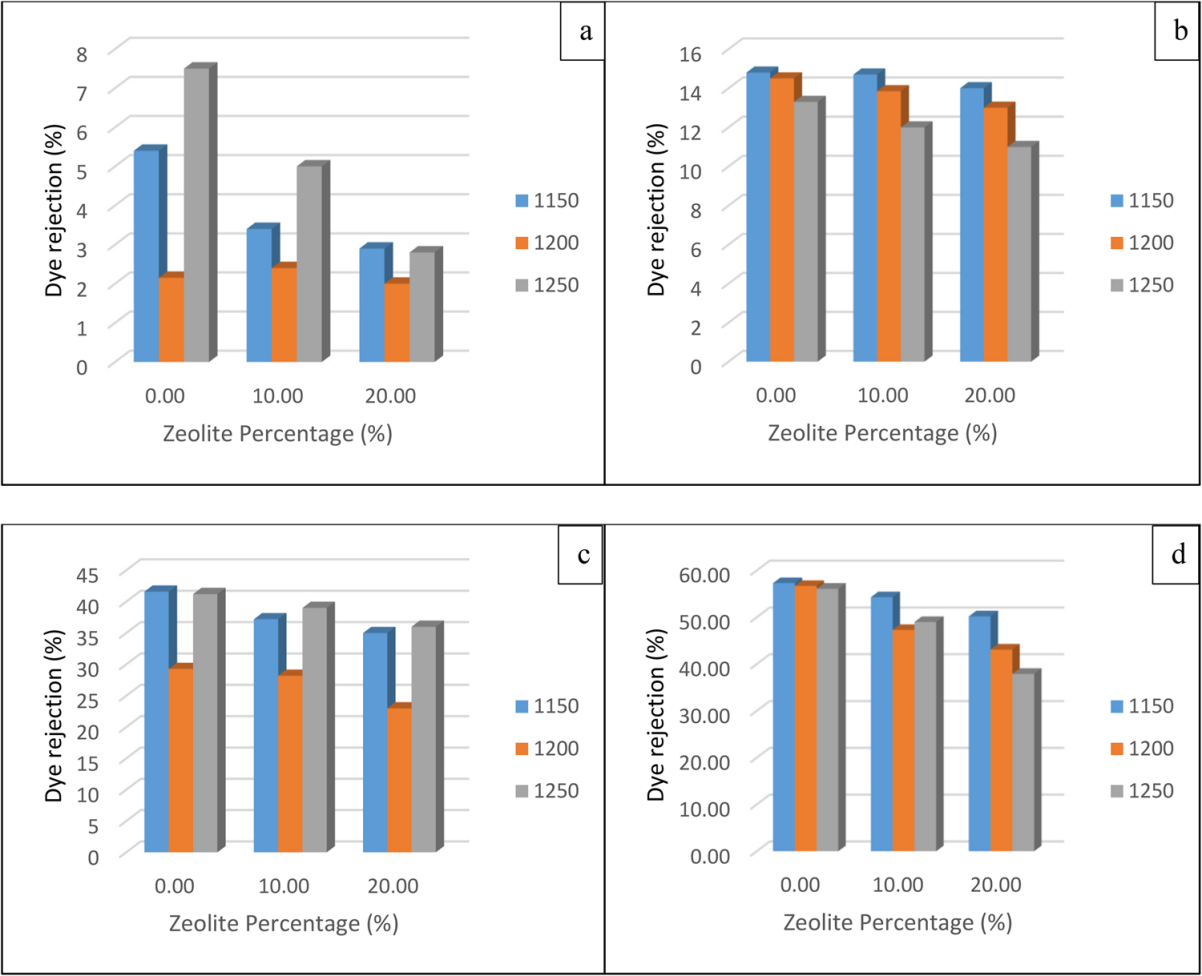
Methylene blue rejection % for soaking times of (**a**) 30 min and (**b**) 60 min and acid fuchsin rejection % for soaking times of (**c**) 30 min and (**d**) 60 min.

Dye removal was calculated for two different dyes, methylene blue (MB) and acid fuchsin (AF), and the conditions and results at 30 and 60 min are shown in Fig. 15. After 30 min of soaking, at temperatures 1150 and 1200, the rejection percentages of MB and AF decreased with temperature due to the increase in pore size; however, at 1250, the densification of the membrane was high enough to reduce the number of pores; thus, the rejection percentage increased. On the other hand, after 60 min of soaking, the rejection percentage decreases with increasing temperature, which shows that increasing the pore size has a greater effect than increasing the densification.

According to previous results, the optimum membrane conditions were selected, and two aspects were taken into consideration: a high-efficiency membrane and low production cost. It was clearly found that increasing the soaking time to more than 30 min had a minimal effect on all the properties and negatively affected the price. An increase in the zeolite percentage sparingly increased some of the properties but affected the membrane production cost due to its high cost. According to the results, a sintering temperature of 1150 °C was chosen because of its good properties in addition to reducing the production cost; therefore, the optimum conditions were 1150 °C and a soaking time of 30 min to achieve a 42% removal of acid fuchsin.

### Modification of the optimum membrane

**Figure 16:**
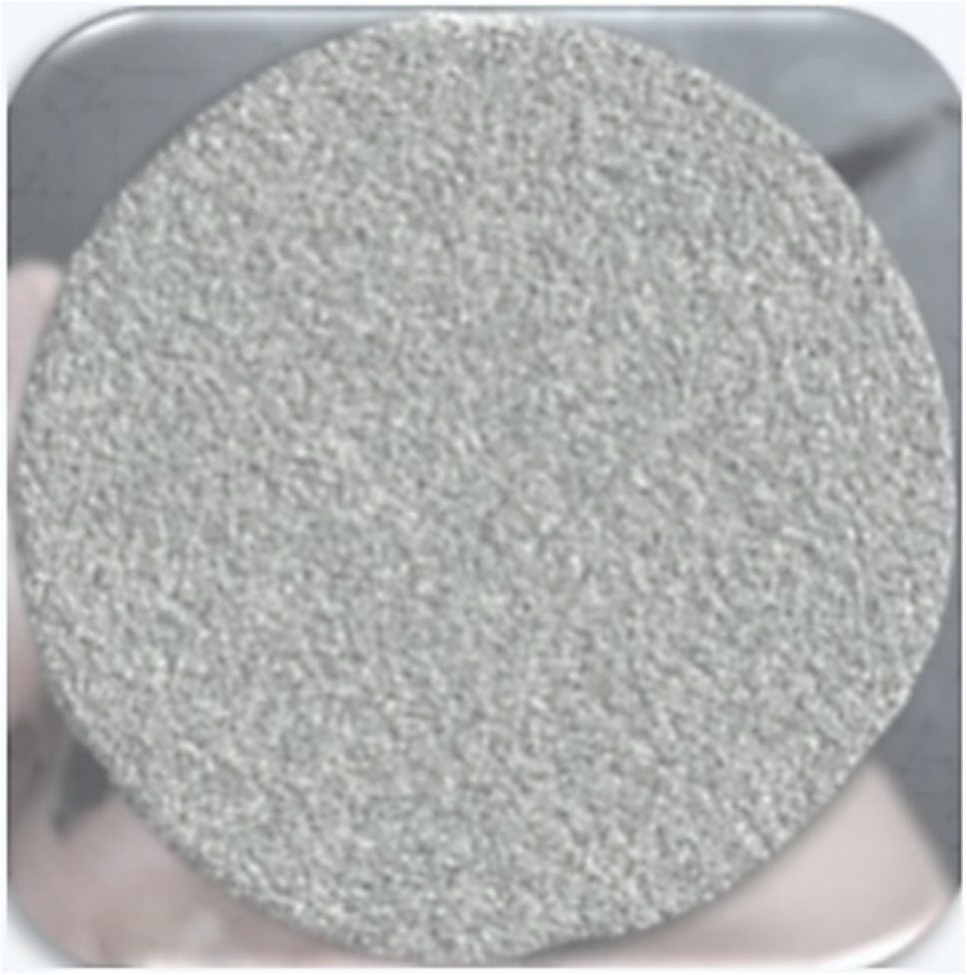
Optimum membrane after surface modification.

The optimum membrane was impregnated with CNTs, a sonication bath was used for homogenous dispersion of the CNTs, and the membrane was then incubated for 1 h; the results are shown in Fig. 16. The rejection percentage of acid fuchsin reached 95%, which was achieved as a result of a decrease in pore size due to the impregnation of CNTs into the pores; moreover, the small diameter of the CNTs aids in the passage of water molecules without contaminants^50^. Several tests were applied to determine the optimum membrane, as described in the following sections.

#### Membrane characterization

##### Membrane pore size

**Figure 17:**
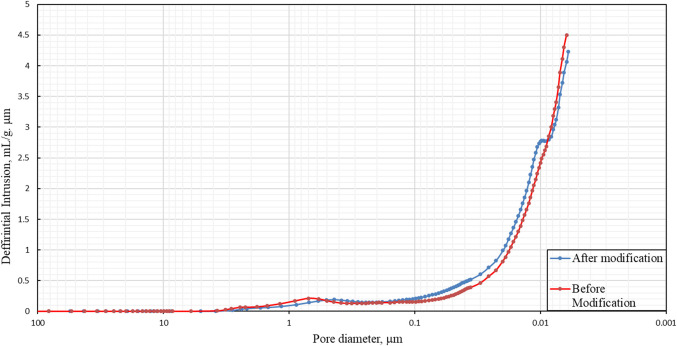
Pore size distribution for the optimum membrane (**a**) before surface modification and (**b**) after surface modification.

The membrane pore size distribution was obtained using a mercury porosimeter, as shown in Fig. 17. The pore size of the membrane ranged from 0.1 to 0.006 μm, and the results for the membrane before and after surface modification are tabulated in Table 3. The average pore diameter is reduced from 0.1 to 0.076 μm by using surface modification, which confirms that the membrane can be used in ultrafiltration applications; on the other hand, the porosity decreases due to the penetration of CNTs into the pores, which is consistent with the SEM results.

**Table 3:** Porosimeter results.

Variable	Membrane before	Membrane after
Total intrusion volume (ml/g)	0.3865	0.3141
Total pore area (m^2^/g)	15.423	16.539
Average pore diameter (micrometers)	0.1000	0.0760
Porosity (%)	52.700	45.800

##### Membrane FTIR before and after CNT treatment

**Figure 18:**
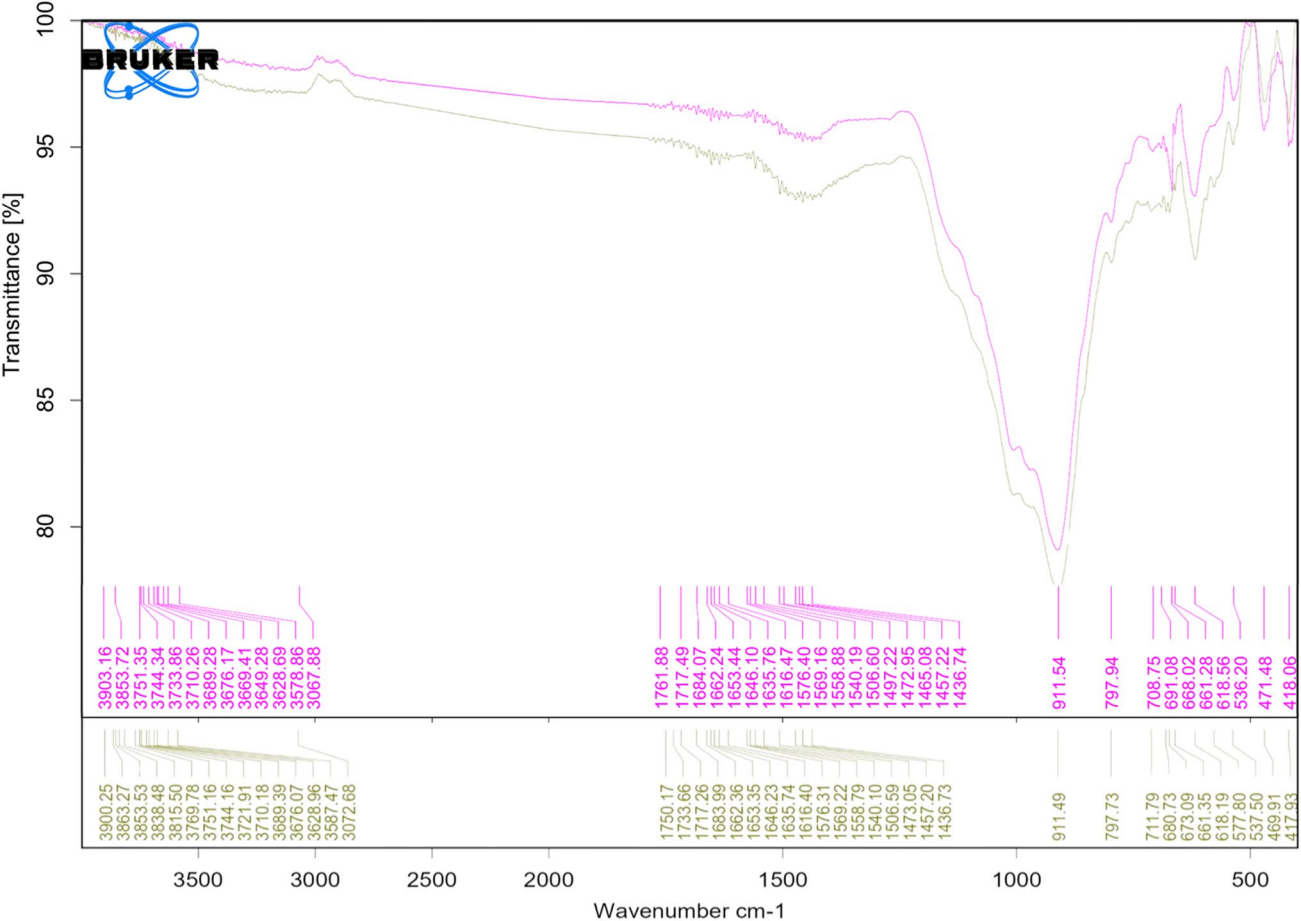
FTIR results of the optimum membrane before and after modification.

The FTIR results of the membrane before and after modification are shown in Fig. 18, all functional groups and their corresponding wave numbers are tabulated in Table 4. The main difference in the wavenumber was between 1733.66 and 1750.17 cm-1 for the membrane after modification, as this range appears due to stretching of C = O in the carboxyl group attached to the carbon nanotubes that was used in surface modification^51^, this confirms the impregnation of CNTs and the stability of membrane.

**Table 4:** FTIR of membrane before and after modification.

Membrane	Wave number, cm^−1^	Functional group
Before modification	3853.72	O–H stretching vibration^52^
	3628.69-3751.35	Al–O–H stretching^44^
	3578.86	stretching vibrations of the OH groups of water molecules^47^
	1635.76-1662.24	bending vibration mode of residual H_2_O molecules^48^
	1616.47	Deformation of OH group^49^
	1436.74-1472.95	Carbonate stretching vibration^53,54^
	911.54	Inner OH bending vibration that caused by Al–OH group^44,47,53^
	691.08 -797.94	stretching vibrations of Si-O-Al^44,53^
	668.02	attributed to the external linkage symmetrical stretching^48,53^
	661.28	bending vibration Si–O^47,48^
	618.56	stretching vibrations of Si-O-Al^47,49,53^
	536.20	Si–O–Al stretching^44,47,53^
	471.48	Si–O–Si bending^44,53^
	418.06	Si–O deformation^54^
After modification	3815.50-3863.27	O–H stretching vibration^52^
	3676.07-3768.78	Al–O–H stretching^44^
	3628.96	Al–O–H stretching^44^
	3587.47	stretching vibrations of the OH groups of water molecules^47^
	3072.68	OH stretching in carboxyl group^52^
	1733.66-1750.17	Stretching of C = O in carboxyl group attached to carbon nanotubes^51^
	1635.74-1662.36	the bending vibration mode of residual H_2_O molecules^48^
	1616.40	Deformation of OH group^49^
	1436.73 -1473.05	Carbonate stretching vibration^43,53,55^
	911.49	Inner OH bending vibration that caused by Al–OH group^44,47,53^
	797.73	Stretching vibrations of Si-O-Al^47,48,53^
	711.79	Si–O–Si (Al) stretching^44,53^
	573.5-680.73	Stretching vibrations of Si-O-Al^47,49,53^
	469.91	Si–O–Si bending^44,53^
	417.93	Si–O deformation^54^

##### Optimum membrane SEM

**Figure 19:**
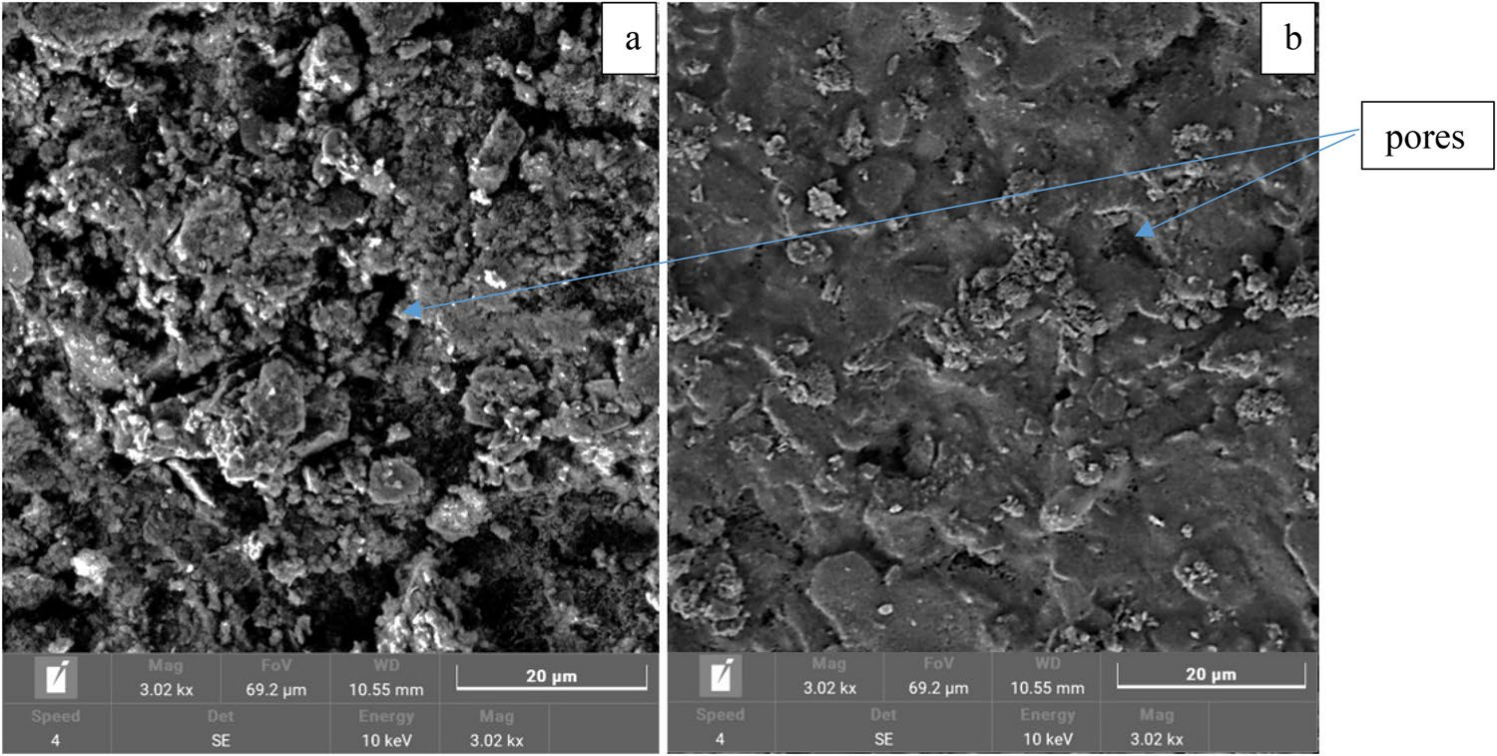
SEM images of the optimal membrane (**a**) before surface modification and (**b**) after surface modification.

SEM was used to scan the microstructure and surface of the optimal membrane, and the results are displayed in Fig. 19.

It is clear that before surface modification, the membrane contained large open pores; however, after modification, the CNTs contributed to closing the pores and generating a membrane with a decreased pore size.

#### Regeneration and anti-fouling analysis

**Figure 20:**
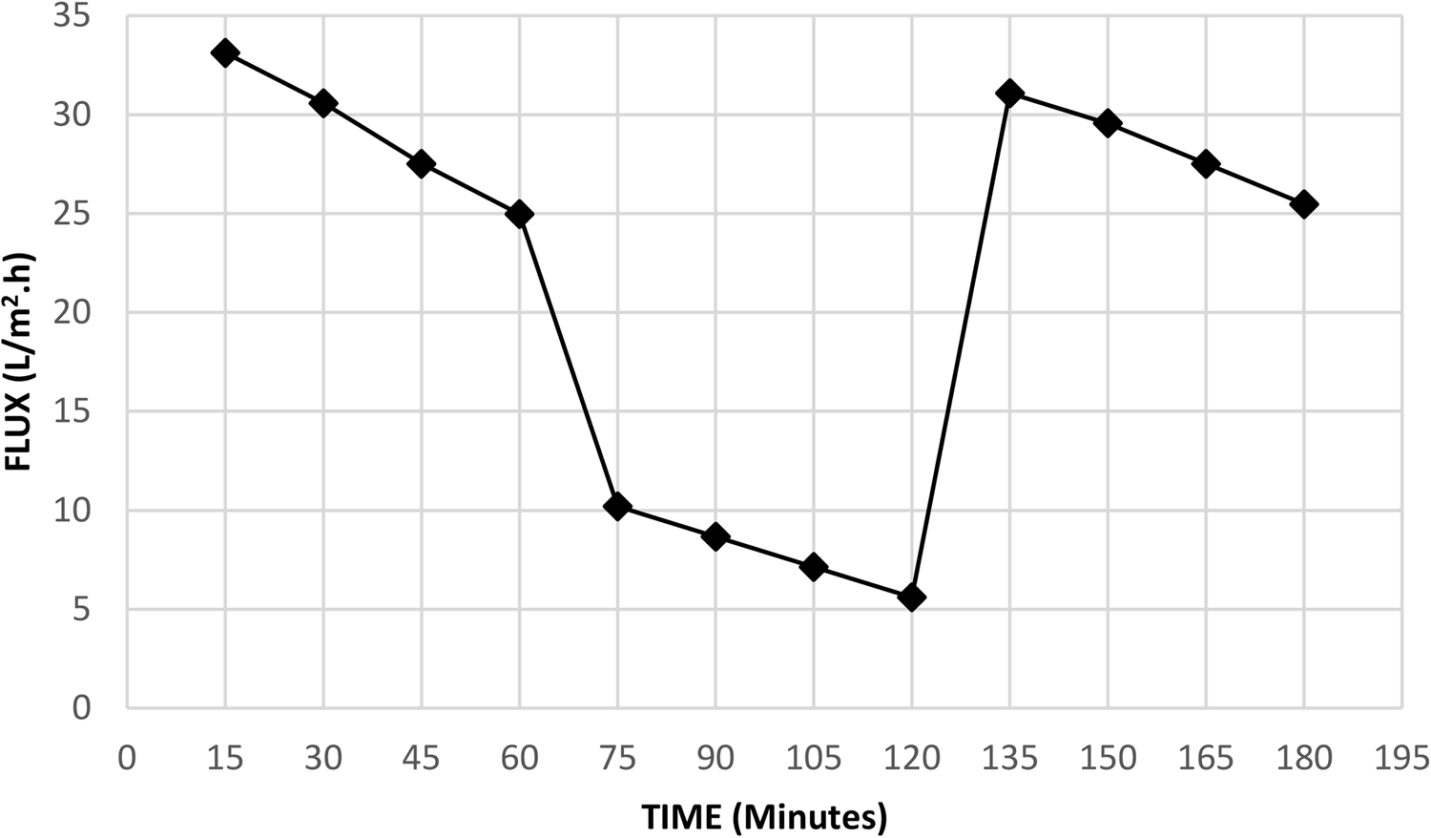
Fouling test results.

The use of functionalized CNTs improves the resistance to fouling due to their negative charge and high hydrophilicity^34^. The fouling of the optimum membrane was studied using a wastewater of 100 PPM acid fuchsin dye and 100 PPM humic acid to simulate real water pollution load, results are shown in Fig. 20. After the filtration process, the membrane become clogged and backwash takes place to restore the permeability of the membrane, it was found that only distilled water can achieve 97.8% cleaning efficiency. Anti-fouling characteristics were studied and results are tabulated in Table 5. The membrane was used for 10 cycles and the rejection decline was only 2% which confirms the effectiveness of demineralized water backwash.

**Table 5:** Anti-fouling characteristics of the optimum membrane.

Item	Value
Total fouling	72.8%
Reversible fouling	70.6%
Irreversible fouling	2.1%
Flux recovery ratio	97.8%

##### Comparative study of the removal percent of dyes using ceramic membranes from literature

Various studies reported the removal efficiency of different dyes using ceramic membranes as shown in Table 6, results confirm the efficiency of the fabricated membrane as its rejection percent is high when compared to previous studies.

**Table 6:** Removal efficiency of different dyes using ceramic membranes from literature.

Acidic dye type	Removal percent (%)
Indigo carmine^56^	98.54
Disperse blue − 79^57^	97
Acid yellow − 23^57^	96.4
Acid orange − 7^57^	95.8
Indigo blue^25^	95
Rhodamine B^58^	80.1
Direct red 80^58^	97
Congo red^59^	95.2

##### Cost estimation and environmental impact

The cost of membrane fabricated in this research has two aspects, the cost of raw materials, and the cost of fabrication. the optimum membrane is fabricated from clay which is inexpensive and available in large quantities, on the other hand the chosen sintering temperature is low, which lower the fabrication cost, the cost of raw materials is tabulated in Table 7, after adding the energy and manpower required, the cost of 1 m^2^ reached about 12 $/m^2^, which is a competitive price compared to literature^60^. The fabricated membrane has minimal environmental impact as it uses available materials, not only that but also the relative low sintering temperature would lower the energy consumption required for production, taking into consideration the long-life expectancy of ceramic membranes, these membranes would contribute positively in the water, food, and energy nexus and as a result in sustainable development.

**Table 7:** Cost analysis of the prepared membranes.

	Unit price ($/kg)	Material required per membrane ($)	Total material cost ($/m^2^)
Clay	0.15	0.0075	
Starch	1.8	0.0045	
PVA	3	0.0008	
CNT-COOH	150	0.0080	
Total material cost		0.0208	10.6

## Conclusion

Industrial wastewater is disposed of in large quantities and has severe negative impacts on the environment. Ceramic membranes are among the leading techniques used in wastewater treatment due to their unique characteristics. A CNT-modified clay-based ceramic membrane was prepared, and its properties and efficiency were assessed according to physical properties and performance. The optimum manufacturing conditions were found to be 1150 °C and a 30-minute soaking time. After impregnating with CNT-COOH, 45.8% of the membrane was porous, with an average pore size of 0.07 micrometers, and the AF dye removal efficiency reached 95%. SEM revealed normally distributed pores. The membranes were subjected to a synthetic wastewater containing 100PPM AF and 100PPM humic acid to study its fouling and performance, membranes exhibited total fouling of 72.8%, of which 2.2% was irreversible fouling, and it was found that demineralized backwash is efficient to achieve cleaning efficiency of 97.8%. The cost analysis of the produced showed a competitive advantage for such a highly efficient, long-lasting membranes, as the cost of 1 m^2^ reached 10.6$ including raw materials and fabrication.

## Data Availability

All data generated or analysed during this study are included in this published article.
